# Within-individual design for assessing true individual responses in resistance training-induced muscle hypertrophy

**DOI:** 10.3389/fspor.2025.1517190

**Published:** 2025-01-31

**Authors:** Talisson Santos Chaves, Deivid Gomes da Silva, Manoel Emílio Lixandrão, Cleiton Augusto Libardi

**Affiliations:** ^1^ MUSCULAB - Laboratory of Neuromuscular Adaptations to Resistance Training, Department of Physical Education, Federal University of São Carlos, São Carlos, São Paulo, Brazil; ^2^ Department of Pediatrics, Section of Nutrition, University of Colorado Anschutz Medical Campus, Aurora, CO, United States

**Keywords:** non responders, typical error of measurement, experimental design, biological vailability, random error analysis

## Abstract

Understanding interindividual variability in muscle hypertrophy in response to resistance training (RT) is a key focus of contemporary research. Common aims include identifying determinants of variability and recognizing individuals who do not respond to RT (i.e., non-responders). However, accurately identifying true individual responses (TIR) remains challenging due to the complex nature of muscle hypertrophy assessments. This complexity arises from distinguishing the TIR from natural variation in muscle mass over time and random measurement error in pre- and postintervention assessments. Existing studies have often overlooked this complexity, failing to employ experimental designs capable of isolating the TIR. Additionally, the reliance on random measurement error assessments based on group level data may not adequately capture the biological variation in muscle mass within individuals. In this context, we propose an experimental design based on unilateral, within-subject resistance training, capable of estimating biological variation in muscle mass and identifying TIR to RT-induced muscle hypertrophy. Additionally, we present an approach to effectively identify non-responders.

## Introduction

Several investigations have focus on understanding the phenomenon of interindividual variability in muscle mass gains (i.e., muscle hypertrophy) induced by resistance training (RT) (1–14). Commonly, the primary aims of these studies are as follows: (1) identifying key determinants of variability in interindividual response (i.e., effect modifiers), to comprehend whether genetic, epigenetic, and environmental/behavioral factors (i.e., diet, sleep, lifestyle, prior experience with RT, and other transient characteristics) are associated, at least in part, for the variability in muscle hypertrophy response among individuals (15); (2) identifying individuals who do not exhibit muscle hypertrophy in response to RT (i.e., non-responders), with the aim of developing strategies that enable these individuals to respond to future interventions (16). Thus, more accurate identification of individual responses appears to be crucial for advancements in understanding interindividual variability.

In this regard, identifying the individual response to muscle hypertrophy can still be considered complex. This is because the observed individual response induced by RT is defined as the sum of the following components (C): C1: change in muscle mass caused by the intervention [i.e., true individual response (TIR)]; C2: change that would have occurred if the individual had not undergone the intervention (i.e., random error resulting from biological variation in the subject's muscle mass over the intervention time); C3: change caused by random measurement error present in pre- and postintervention assessments (i.e., random error resulting from the measurement technique and the evaluator). Thus, considering the existence of C2 and C3, the changes in muscle mass observed in an individual after an RT program may or not be attributed to the intervention itself. Therefore, accurate identification of the TIR would involve isolating C1 from the other sources of variation (C2 and C3).

Studies that investigated the individual responses to muscle hypertrophy did not use any experimental design strategy or analysis to isolate the TIR from all other sources of variation simultaneously (i.e., C2 and C3) (1–14). Ahtiainen et al. (9), did indeed examine the long-term changes in muscle size within a control group. However, the authors refrained from incorporating the observed variation within the control group when considering the estimate of the adaptive response to RT within the intervention group. Despite this, we recognize that some studies that investigated individual responses considered partially the influence of these sources of variation using random measurement error consisting of two repeated measures, which includes technical error and short-term day-to-day variability in measurements (12, 14, 17). However, we emphasize that variation observed in a short period (e.g., 72 h or one week) may not fully capture biological variation in muscle mass within an individual who occurs throughout the entire intervention. Furthermore, random measurement error was assessed based on test-retest data from a group of individuals. In the context of individual responses, this can be problematic because although the random measurement error may be low and consistent among most individuals in the sample (12, 14, 17), the biological variation in muscle mass within individuals over time may be high and differ substantially between subjects, especially when the follow-up time is long (e.g., 3–6 months) (18). In this case, the random error performed with a short period in a group of individuals may not be a valid measure of the biological variation in muscle mass for an individual. Therefore, to more precisely identify TIRs, improve the effectiveness of analyzing effect modifiers and identify non-responders, individualized measures of C2 are needed.

Thus, this study proposes an analysis based on an experimental design that allows to estimate the natural variation in an individual's muscle mass over the intervention period and, consequently, estimates the TIR in RT-induced muscle hypertrophy. Additionally, we propose an approach to identify non-responders.

## Quantifying the magnitude of interindividual variability: the need for a control group

Before analyzing effect, modifiers or identifying non-responders, it has been suggested that the true interindividual variability (i.e., attributable only to the intervention) be quantified (19, 20). In this context, to understand the magnitude of interindividual variability, it is necessary to analyze the standard deviation (SD) associated with the average response of a group after an intervention (SD*int*). Indeed, the SD*int* represents the variation in individual responses around the group mean (20). However, SD*int* is composed of TIRs, accompanied by their sources of random error (19). The presence of random errors can overestimate or underestimate the true interindividual variability of the response. To minimize this issue, it has been suggested that experimental designs include a parallel control group (i.e., accompanied for the same period as the intervention group) (15, 19, 20).

The reason for the inclusion of a control group is based on two assumptions: (1) changes in the SD of the control group (SD*con*) estimate the interindividual variability due to random error alone; (2) the variability due to random error is similar between control and intervention groups, differing only in the variability caused by the intervention. Thus, if both assumptions are met, such an experimental design allows us to estimate the true interindividual variability (i.e., SD*true*), by simply subtracting the variance in the control group from the variance observed in the intervention group (20). Therefore, the SD*true* represents a measure of the variability of individual responses around the mean group effect, adjusted for the influence of random error, from Equation 1a (the description of the equation can be found in the Supplementary File).

(1a) $$SDtrue\;=\;\sqrt{\left(SDint^{2}-SDcon^{2}\right)}$$

Although parallel control group accounts for the magnitude of variability within the intervention group, adjusted for the influence of the random error this method has limitations for estimating the TIR for an individual (21, 22). This occurs because the observed individual hypertrophic response due to the intervention must be adjusted for biological variation in muscle mass that occurs throughout within the same period, but without any intervention. Unfortunately, the use of independent groups makes this procedure impossible as individuals are either allocated to the intervention or control group only, thus it is impossible to quantify its biological variation in different experimental conditions at the same time. Even if a control period of equal duration were conducted prior to the intervention, where each subject would serve as their own control parameter, environmental and behavioral factors could vary throughout the experiment. These variations might affect individuals differently during the control and intervention periods.

Therefore, we suggest that an experimental design, where everyone is used as their own control throughout the intervention (further details below), can minimize all limitations mentioned thus far when investigating muscle hypertrophy.

## Within-person trial design as an ideal approach to assess individual responses to muscle hypertrophy

Estimating the TIR in muscle mass gains after intervention is challenging. Here, we suggest a within-individual trial design, in which the randomization unit is not the individual but rather an organ or some part of the body (23, 24). This design allows to carry out an intervention in one of the participants' limbs, while the contralateral limb does not undergo any intervention throughout the entire experimental period (i.e., control condition). The use of the contralateral limb as a control in RT studies has been employed to estimate natural variation in muscle mass over time (1, 25). However, our approach introduces novelty by combining the calculation of the SD*true* using both the intervention and control limbs, the analysis that allows quantification of C2 and, based on this, estimates TIR with greater precision for each individual., and the use of 95% CI based on measurement error around TIR as a suggested predefined criterion for identifying non-responders.

## Estimating the magnitude of true interindividual variability in muscle hypertrophy

The use of a within-subject experimental design in which one leg is assigned to a RT program while the contralateral leg serves as an internal control assumes that changes in the untrained leg estimates the random error arising from biological variations in muscle mass over the experimental period (i.e., C2). In this case, researchers can measure changes in muscle mass for both limbs (exercised and control) and their respective standard deviation (i.e., SD*int* and SD*cont*) within the same individual. This information allows us to calculate the magnitude of true interindividual variability as suggested by previous studies. For example, in a within-subject study conducted by Hubal et al. (1), RT was performed only in the non-dominant arm and the untrained contralateral limb was used as an internal control. However, the results for the untrained limb were not used for an analysis of interindividual variability. To verify the influence of C2 and C3 in Hubal's study, we considered the mean and SD of the percentage change in muscle mass for the 12-week trained limb (18.9 ± 9.7%) and for the untrained limb (1.4 ± 7.2%). The SD of the RT group *a priori* indicates an interindividual variability of 9.7%. However, through the differences between the variances of trained and untrained limbs, we found that the true standard deviation (SD*true*) from Equation 1b (the description of the equation can be found in the Supplementary File).

(1b) $$SDtrue=\;\sqrt{\left(9.7^{2}-7.2^{2}\right)}=6.5\text{\%}$$

Clearly, this indicates an approximately 33% decrease in interindividual variability due to intervention. Therefore, this analysis highlights that interindividual variability was reduced when accounting for changes in the control group in the analysis. It is important to note that both parallel-group and within-subject designs can capture the true interindividual variability present in the group. However, the within-subject design may be more advantageous as it enables researchers to control potential behavioral/environmental confounding factors (e.g., diet, sleep, training history). In addition, the use of a within-person trial design reduces by half the number of subjects needed to carry out the study, which is particularly important in view of the difficulty of obtaining a large sample size for long-term interventions, as well as the financial costs of the project. Thus, we suggest that future studies use this approach to identify the magnitude of true interindividual variability in muscle mass gains.

## Estimating the true individual response to muscle hypertrophy

Training only one of the limbs, while the contralateral is used as control, allows estimating the magnitude of the true interindividual variability and, therefore, the variation resulting from the C2 for everyone. Considering the influence of this variation on the observed response in the limb undergoing intervention, it leads to the estimation of the TIR. This is crucial because while C3 may be low and consistent in group analyses, based on reproducibility metrics over a short inter-measurement interval, the error associated with biological variation in muscle mass estimated from control group analysis can significantly vary between subjects, particularly with longer follow-up period (i.e., C2) (e.g., 3–6 months) (19). Additionally, C2 is often greater than C3 (26); therefore, this source of variation can be considered amongst the main obstacle in estimating the TIR to muscle hypertrophy induced by RT in non-within-subject approaches.

Thus, based on the premise that randomized controlled trials estimate the intervention effect, by subtracting changes in intervention from control groups, the within-subject design proposed herein allows estimating the TIR. That is, researchers can subtract the response observed in the trained limb from the observed response of the control one (i.e., *Δ_post−pre_*trained limb – *Δ_post−pre_* control limb). For example, if an individual exhibits a 12% increase in the muscle cross-sectional area of the trained limb and a 5% positive variation in the control leg, the TIR to the intervention is 7%. This approach allows for estimating the magnitude of variation in C2 and considering its influence on the individual response. Thus, it enables only the estimates of TIR to be considered for effect modifier analyses. In this case, by mitigating the within-group variability caused by potential confounding factors, it is expected that the power of the statistical tests used in the analysis of effect modifiers (e.g., linear regression) will increase (27, 28). In other words, such a procedure can facilitate the detection of possible associations between the dependent variable (i.e., muscle hypertrophy) and potential effect-modifying variables if they exist (e.g., diet, sleep, lifestyle, training history and other transient characteristics). Therefore, such an approach can help researchers explore the effect of modifying variables that contribute to the variability in muscle hypertrophy responses, potentially guiding future investigations into these modifiers.

## Identifying non-responders for muscle hypertrophy

The identification of non-responders has been suggested as an important step in understanding interindividual variability in muscle mass gains. However, separating study participants into “responders” and “non-responders” can be problematic if objective, predefined criteria are not established *a priori*, as it often serves as a device to assert that some subset of the sample experienced a beneficial effect from the intervention, even when the average treatment effect is small or not significant. This approach tends to overlook possible moderators and mediators, which would be more scientific ways to explain why the intervention or treatment results in heterogeneous responses. When there is heterogeneity in response, the most appropriate approach is to investigate the likely mechanisms, causal models, and other ways to quantify this heterogeneity (29–31). On the other hand, researchers may explore the efficacy of alternative training interventions and dietary or pharmacological options for individuals who exhibit little or no response (16).

To potentially minimize bias in identifying non-responders, it is necessary to control for the influence of C2 and C3 (i.e., biological variation in muscle mass over the intervention period + error from measurement technique and evaluator) on the observed response after the intervention. As described above, the TIR estimates the individual response by accounting for the influence of potential biological variation in muscle mass observed in the contralateral control limb. The next step is to consider the influence of measurement error in identifying non-responders. For this purpose, we suggest including a confidence interval (CI) composed of the measurement error (i.e., typical error [TE] or coefficient of variation [CV]) (18, 20, 30, 31) (the description of the TE and CV can be found in the Supplementary File). Around the TIR, we suggest that the TE be calculated based on 2 or more repeated measurements performed on all individuals with a short interval between measurements. Thus, to calculate the 95% CI based on C3, the choice between TE or CV depends on whether the measured variable is reported in absolute or relative values, respectively (18). Equation 2a was utilized (the description of the equation can be found in the Supplementary File).

(2a) $$95\text{\%}\;\mathrm{CI}\;=\;1.96\;\times \;\sqrt{2}\times \;\mathrm{CV}\;$$

Considering that inaccuracies can occur both above and below the TIR, the value produced by the formula is added and subtracted from the TIR, creating a 95% CI around this estimate. To illustrate, if the estimated TIR after an intervention with RT for an individual is 7% and the CV of the measure is 1%, we can calculate the 95% CI From Equation 2b:

(2b) $$95\text{\%}\;\mathrm{CI}\;=\;1.96\;\times \;\sqrt{2}\times \;1\;=\;2.77\text{\%}\;$$

**Figure 1:**
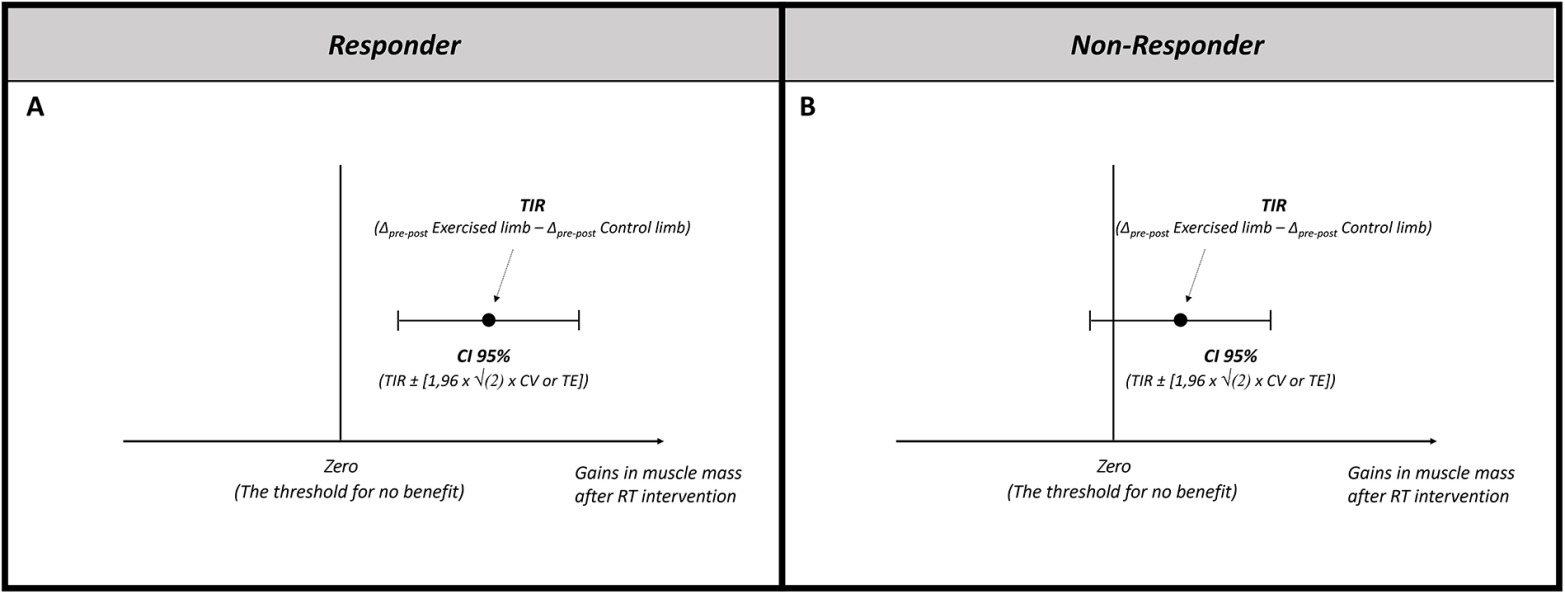
Identifying non-responders for muscle hypertrophy following resistance training. **(A)** A responder is considered when the lower bound of the 95% CI is above the threshold that represents no benefit (i.e., zero). **(B)** A non-responder is considered when the 95% CI includes the threshold that represents no benefit (i.e., zero). TIR, true individual response; CI, confidence interval; CV, coefficient of variation; TE, typical error.

Thus, the range of values for TIR would be TIR = 7% (95% CI = 4.23%; 9.77%). Therefore, the CI around the observed response represents a range of plausible values for the TIR, accounting for the imprecision of the muscle mass assessment method (i.e., measurement error). In this sense, although the best estimate for the individual's response is 7%, it cannot be ignored that due to C3, the TIR would be between 4.2 and 9.6%. Therefore, RT interventions can be classified as successful or unsuccessful for each individual if the 95% CI around the TIR is within a predefined region (18). For example, the intervention can be considered effective for a given individual if the lower bound of the 95% CI around the TIR for muscle mass is above the threshold that represents no benefit (i.e., zero). In contrast, if the 95% CI includes zero or if the upper limit of this range was below zero, the subject can be classified as non-responder (Figure 1). A more detailed explanation of the construction and use of CIs based on measurement error is beyond the scope of this article, and readers should refer to Swinton et al. (18). We consider this approach to be adequate for identifying non-responders to muscle hypertrophy, as the within-person design allows the estimation of C2 within everyone throughout the intervention period. Further, we emphasize that by subtracting this source of variation, the TIR may differ substantially from the initially observed response (i.e., without adjustment for random error). Consequently, the proposed approach can have a substantial impact on the number of non-responders observed in studies.

## Limitations

While our proposed approach provides a structured framework for estimating TIR to RT-induced hypertrophy by accounting for biological variation and measurement error, methodological limitations should be acknowledged. Firstly, the design is restricted to RT protocols involving isolation movements and specific muscle groups, primarily the limbs. Consequently, its applicability to multi-joint, compound exercises and whole-body RT adaptations may be limited. Additionally, this method is primarily suited for assessing muscle mass changes, as it does not account for neural adaptations or performance outcomes such as maximal strength, power, or motor skill acquisition. This limitation arises partly due to the cross-education phenomenon, where training one limb can influence performance improvements in the untrained limb. Future research could consider integrating additional assessments to capture a broader spectrum of physiological adaptations.

## Conclusion

We proposed an analysis based on a within-subject experimental design, which enables the estimation of biological variation in an individual's muscle mass over the intervention period. This approach allows the estimation of the TIR to RT-induced muscle hypertrophy. Consequently, accurately estimating the TIR can enhance investigations dedicated to analyzing effect modifiers. Furthermore, it enables the more appropriate identification of non-responders to RT interventions.

## Data Availability

The original contributions presented in the study are included in the article/Supplementary Material.

## References

[1] Hubal MJ Gordish-Dressman H Thompson PD Price TB Hoffman EP Angelopoulos TJ Variability in muscle size and strength gain after unilateral resistance training. Med Sci Sports Exerc. (2005) 37(6):964–72. 15947721

[2] Bamman MM Petrella JK Kim JS Mayhew DL Cross JM. Cluster analysis tests the importance of myogenic gene expression during myofiber hypertrophy in humans. J Appl Physiol (1985). (2007) 102(6):2232–9. 10.1152/japplphysiol.00024.2007 17395765

[3] Kim JS Petrella JK Cross JM Bamman MM. Load-mediated downregulation of myostatin mRNA is not sufficient to promote myofiber hypertrophy in humans: a cluster analysis. J Appl Physiol (1985). (2007) 103(5):1488–95. 10.1152/japplphysiol.01194.2006 17673556

[4] Petrella JK Kim JS Mayhew DL Cross JM Bamman MM. Potent myofiber hypertrophy during resistance training in humans is associated with satellite cell-mediated myonuclear addition: a cluster analysis. J Appl Physiol (1985). (2008) 104(6):1736–42. 10.1152/japplphysiol.01215.2007 18436694

[5] Thalacker-Mercer AE Petrella JK Bamman MM. Does habitual dietary intake influence myofiber hypertrophy in response to resistance training? A cluster analysis. Appl Physiol Nutr Metab. (2009) 34(4):632–9. 10.1139/H09-038 19767798 PMC3188961

[6] Erskine RM Jones DA Williams AG Stewart CE Degens H. Inter-individual variability in the adaptation of human muscle specific tension to progressive resistance training. Eur J Appl Physiol. (2010) 110(6):1117–25. 10.1007/s00421-010-1601-9 20703498

[7] Davidsen PK Gallagher IJ Hartman JW Tarnopolsky MA Dela F Helge JW High responders to resistance exercise training demonstrate differential regulation of skeletal muscle microRNA expression. J Appl Physiol (1985). (2011) 110(2):309–17. 10.1152/japplphysiol.00901.2010 21030674

[8] Thalacker-Mercer A Stec M Cui X Cross J Windham S Bamman M. Cluster analysis reveals differential transcript profiles associated with resistance training-induced human skeletal muscle hypertrophy. Physiol Genomics. (2013) 45(12):499–507. 10.1152/physiolgenomics.00167.2012 23632419 PMC3680779

[9] Ahtiainen JP Walker S Peltonen H Holviala J Sillanpaa E Karavirta L Heterogeneity in resistance training-induced muscle strength and mass responses in men and women of different ages. Age (Dordr). (2016) 38(1):10. 10.1007/s11357-015-9870-1 26767377 PMC5005877

[10] Mobley CB Haun CT Roberson PA Mumford PW Kephart WC Romero MA Biomarkers associated with low, moderate, and high vastus lateralis muscle hypertrophy following 12 weeks of resistance training. PLoS One. (2018) 13(4):e0195203. 10.1371/journal.pone.0195203 29621305 PMC5886420

[11] Morton RW Sato K Gallaugher MPB Oikawa SY McNicholas PD Fujita S Muscle androgen receptor content but not systemic hormones is associated with resistance training-induced skeletal muscle hypertrophy in healthy, young men. Front Physiol. (2018) 9:1373. 10.3389/fphys.2018.01373 30356739 PMC6189473

[12] Damas F Barcelos C Nobrega SR Ugrinowitsch C Lixandrao ME Santos L Individual muscle hypertrophy and strength responses to high vs. Low resistance training frequencies. J Strength Cond Res. (2019) 33(4):897–901. 10.1519/JSC.0000000000002864 30289872

[13] Haun CT Vann CG Mobley CB Osburn SC Mumford PW Roberson PA Pre-training skeletal muscle fiber size and predominant fiber type best predict hypertrophic responses to 6 weeks of resistance training in previously trained young men. Front Physiol. (2019) 10:297. 10.3389/fphys.2019.00297 30971942 PMC6445136

[14] Nunes JP Pina FLC Ribeiro AS Cunha PM Kassiano W Costa BDV Responsiveness to muscle mass gain following 12 and 24 weeks of resistance training in older women. Aging Clin Exp Res. (2021) 33(4):1071–8. 10.1007/s40520-020-01587-z 32447738

[15] Ross R Goodpaster BH Koch LG Sarzynski MA Kohrt WM Johannsen NM Precision exercise medicine: understanding exercise response variability. Br J Sports Med. (2019) 53(18):1141–53. 10.1136/bjsports-2018-100328 30862704 PMC6818669

[16] Williamson PJ Atkinson G Batterham AM. Inter-individual responses of maximal oxygen uptake to exercise training: a critical review. Sports Med. (2017) 47(8):1501–13. 10.1007/s40279-017-0680-8 28097487

[17] Lixandrao ME Bamman M Vechin FC Conceicao MS Telles G Longobardi I Higher resistance training volume offsets muscle hypertrophy nonresponsiveness in older individuals. J Appl Physiol (1985). (2024) 136(2):421–9. 10.1152/japplphysiol.00670.2023 38174375

[18] Swinton PA Hemingway BS Saunders B Gualano B Dolan E. A statistical framework to interpret individual response to intervention: paving the way for personalized nutrition and exercise prescription. Front Nutr. (2018) 5:41. 10.3389/fnut.2018.00041 29892599 PMC5985399

[19] Atkinson G Batterham AM. True and false interindividual differences in the physiological response to an intervention. Exp Physiol. (2015) 100(6):577–88. 10.1113/EP085070 25823596

[20] Hopkins WG. Individual responses made easy. J Appl Physiol (1985). (2015) 118(12):1444–6. 10.1152/japplphysiol.00098.2015 25678695

[21] Hecksteden A Kraushaar J Scharhag-Rosenberger F Theisen D Senn S Meyer T. Individual response to exercise training—a statistical perspective. J Appl Physiol (1985). (2015) 118(12):1450–9. 10.1152/japplphysiol.00714.2014 25663672

[22] Dankel SJ Loenneke JP. A method to stop analyzing random error and start analyzing differential responders to exercise. Sports Med. (2020) 50(2):231–8. 10.1007/s40279-019-01147-0 31254258

[23] MacInnis MJ McGlory C Gibala MJ Phillips SM. Investigating human skeletal muscle physiology with unilateral exercise models: when one limb is more powerful than two. Appl Physiol Nutr Metab. (2017) 42(6):563–70. 10.1139/apnm-2016-0645 28177712

[24] Pandis N Chung B Scherer RW Elbourne D Altman DG. CONSORT 2010 Statement: extension checklist for reporting within person randomised trials. Br J Dermatol. (2019) 180(3):534–52. 10.1111/bjd.17239 30609010

[25] Dias NF Bergamasco JGA Scarpelli MC Silva DG Chaves TS Bittencourt D Changes in muscle cross-sectional area during two menstrual cycles may not be exclusively attributed to resistance training. Appl Physiol Nutr Metab. (2024) 49(12):1729–39. 10.1139/apnm-2024-0004 39303293

[26] Atkinson G Nevill AM. Statistical methods for assessing measurement error (reliability) in variables relevant to sports medicine. Sports Med. (1998) 26(4):217–38. 10.2165/00007256-199826040-00002 9820922

[27] Norton BJ Strube MJ. Understanding statistical power. J Orthop Sports Phys Ther. (2001) 31(6):307–15. 10.2519/jospt.2001.31.6.307 11411625

[28] Lazic SE. Four simple ways to increase power without increasing the sample size. Lab Anim. (2018) 52(6):621–9. 10.1177/0023677218767478 29629616

[29] Senn S. Individual response to treatment: is it a valid assumption? Br Med J. (2004) 329(7472):966–8. 10.1136/bmj.329.7472.966 15499115 PMC524113

[30] Senn S Rolfe K Julious SA. Investigating variability in patient response to treatment–a case study from a replicate cross-over study. Stat Methods Med Res. (2011) 20(6):657–66. 10.1177/0962280210379174 20739334

[31] Atkinson G Williamson P Batterham AM. Issues in the determination of ‘responders’ and ‘non-responders’ in physiological research. Exp Physiol. (2019) 104(8):1215–25. 10.1113/EP087712 31116468

