# Supplementary material for: Within-individual design for assessing true individual responses in resistance training-induced muscle hypertrophy
Source: Front Sports Act Living. 2025 Jan 31;7:1517190. doi: 10.3389/fspor.2025.1517190 (PMC11825802; doi:10.3389/fspor.2025.1517190)
Supplement: Supplementary file 1 [file Table1.docx]

**Supplemental file**

**List of Equations**

$$SDtrue = \sqrt{({SDint}^{2}-{SDcon}^{2})} (1)$$

Equation 1. SDint represents the standard deviation observed in the intervention group. SDcon represents the standard deviation observed in the control group. SDtrue represents the estimate of the true standard deviation, considering the variability of the intervention group and the control group.

$$95\% CI = 1.96 \times\sqrt{2}\times CV (2)$$

Equation 2. The 95% confidence interval (95% CI) represents the estimate of the confidence interval with a 95% probability of containing the true value. The constant 1.96 represents the critical z-value for a 95% confidence interval. √2 is the constant used to account for uncertainty in repeated measurements when there are 2 observations. When there are more than two repeated measurements (e.g., 3 or 4), the formula must be adjusted to reflect the increased number of observations. For instance, for 3 repeated measurements, the adjusted constant becomes √3, and for 4, √4. It is crucial that this adjustment is performed consistently with the formula used to determine the typical error of measurement (TE). CV is the coefficient of variation based on the typical measurement error.

$TE =\frac{SDdiff\left( T1 - T2 \right)}{\sqrt{2}} (3)$

Equation 3: In this equation, TE represents the estimate of the typical error of measurement, reflects the variation in the measurement unit that occurs when the same individual is assessed repeatedly under the same conditions. SDdiff(T1-T2) refers to the standard deviation of the mean difference between the values recorded at T1 and T2 for each participant. The constant √2 is used to adjust the typical error estimate due to the use of repeated measures at T1 and T2. In this context, T1 corresponds to the time of assessment at pre-test 1, while T2 refers to the time of assessment at pre-test 2.

$CV =\frac{TE}{Mean\left( T1; T2 \right)}x 100 (4)$

Equation 4. The typical measurement error (TE) reflects the variation in the measurement unit that occurs when the same individual is assessed repeatedly under the same conditions. The Coefficient of Variation (CV), on the other hand, is the percentage representation of the typical measurement error (TE), meaning it expresses this variation as a percentage of the mean of the observed values, rather than using the original measurement unit. For this reason, TE appears as the numerator in the CV formula. The mean (T1; T2) represents the average of the values observed at time points T1 and T2. T1 corresponds to the assessment time at pre-1, and T2 corresponds to the assessment time at pre-2.
